# Exploratory research on the application of the innovative 7E learning cycle model - ideological and political education in curriculum in otorhinolaryngology nursing student teaching

**DOI:** 10.1186/s12909-026-10099-9

**Published:** 2026-08-13

**Authors:** Hongying Xiao, Xiaofang Chen, Rong Chen, Mengting Zhu

**Affiliations:** 1 Deyang People’s Hospital, Deyang, 618000 China; 2 Chengdu University, Chengdu, 610000 China

**Keywords:** 7E Learning Cycle Model, Ideological and Political Education in Curriculum

## Abstract

**Objective:**

To explore the effectiveness of the innovative “7E Learning Cycle Model - Ideological and Political Education in Curriculum” approach in otorhinolaryngology nursing student teaching.

**Methods:**

From April 2024 to August 2025, nursing students interning in our department were selected as the research subjects. This quasi-experimental study enrolled a total of 86 participants with 43 cases in the experimental group receiving the “7E + Ideological and Political Education” teaching method, and 43 cases in the control group receiving traditional teaching methods. Teaching effectiveness was comprehensively evaluated through theoretical assessments, skill assessments, ideological and political literacy scores, and questionnaires.

**Results:**

The experimental group performed significantly better than the control group in terms of professional knowledge, operational skills, ideological and political literacy, and even course satisfaction (*P* < 0.05). Students’ reflective feedback from the experimental group suggested that case discussions and situational simulations effectively facilitated the integration of ideological and political elements such as “humanistic care” and “medical ethics,” encouraging active reflection on the meaning of the nursing profession.

**Conclusion:**

The “7E Learning Cycle - Ideological and Political Education in Curriculum” model contributes to enhancing the effectiveness of clinical internship teaching in otorhinolaryngology, improves nursing students’ self-directed learning ability, empathy, and ideological-political literacy, and enhances their communication skills and attitudes.

## Introduction

With the continuous advancement of the Healthy China strategy, nursing education is increasingly emphasizing the cultivation of high-quality talent. Clinical internship is a critical transitional stage in which nursing students apply theoretical knowledge to authentic practice [1], and the teaching model directly impacts the development of their professional competence. Many clinical teaching methods still predominantly follow a teacher-centered approach, where instructors lecture and students passively receive information [2]. This approach results in students’ passive learning, limited room for autonomous exploration, and challenges in integrating values education meaningfully into professional training. Although active learning approaches such as problem based learning (PBL), clinical simulation and flipped classroom have been verified to boost nursing students’ critical thinking [3–6], students still lack sufficient self-directed learning motivation, clinical empathy, humanistic care awareness and communication skills in real clinical settings. These shortcomings of existing teaching models fail to meet the developmental requirements of modern nursing education, which demands nursing talents to possess not only proficient professional skills, but also sound professional ethics, humanistic qualities and professional responsibility [7]. In this context, integrating ideological and political education in curriculum into nursing teaching has become an inevitable trend, making it necessary to explore an optimized integrated teaching model to address the deficiencies of current clinical nursing teaching.

First proposed in Shanghai in 2014 [8], the concept of “Ideological and Political Education in Curriculum” constitutes a standardized holistic educational framework, which integrates moral cultivation and value guidance into professional and general courses [9], rather than relying on standalone ideological and political courses. As specified in 《Guideline for Ideological and Political Education of Nursing Professional Courses》 [10], nursing ideological and political education in curriculum commits to cultivating students’ comprehensive professional literacy and the medical spirit of respecting life, healing the wounded and rescuing the dying, dedication, and universal benevolence. It prioritizes human life safety and physical health, aiming to cultivate trustworthy and competent nursing practitioners. This framework aligns with internationally recognized nursing education goals. It cultivates professionalism and ethical reasoning by integrating professional ethics into clinical teaching; fosters humanistic care by emphasizing patient-centered communication and empathy; develops social responsibility by encouraging students to consider the broader societal impact of nursing practice; and consolidates professional identity by instilling core professional values across the curriculum. In this way, ideological and political education in curriculum can be seen as a locally developed but globally meaningful approach to addressing the universal challenge of holistic nursing education.

Current ideological and political education in nursing curricula is often limited by rigid formats and disconnection from clinical contexts, making it difficult to elicit students’ emotional engagement or professional value recognition [11]. The 7E learning cycle model, based on constructivist theory, consists of seven stages: Elicit, Engage, Explore, Explain, Elaborate, Evaluate, and Extend [12]. It emphasizes students’ active exploration and autonomous knowledge construction in authentic contexts, with well-defined stage-specific objectives that allow teachers to adjust teaching methods flexibly. The model’s progressive design guides students from basic cognition and independent inquiry toward deeper understanding and practical application within clinical scenarios [13, 14]. Through this step-by-step process, students gradually internalize the professional values that shape their nursing identity. Thus, the model’s phased structure provides a natural scaffold for embedding humanistic care, ethical reasoning, and professional identity formation into routine clinical teaching. Previous studies have demonstrated the effectiveness of the 7E model in improving nursing students’ critical thinking, self-efficacy, and clinical practical competence [14–16]. However, existing research has predominantly focused on cognitive and skill-based outcomes, with little attention paid to the integration of the 7E model with ideological and political education, particularly regarding its effects on nursing students’ professional identity, humanistic care awareness, and ethical reasoning.

Otorhinolaryngology is a highly specialized discipline with a wide variety of diseases, placing high demands on nursing students’ comprehensive abilities and emergency coping skills [17]. Nursing students majoring in this discipline are required to master solid clinical skills and core professional literacy, including patient-centered service awareness, ethical judgment ability under clinical pressure, and empathic communication competence when treating patients with sensory dysfunction. Therefore, this study integrates the 7E learning cycle model with ideological and political education in curriculum and implements it in the clinical teaching of otorhinolaryngology. The aim is to enhance students’ professional proficiency and strengthen their humanistic care awareness and professional values. It is hoped that this exploration will bring new inspiration to nursing clinical teaching reform and provide a replicable model for integrating values education into clinical training.

## Methods

### Participants and study design

A quasi-experimental design was adopted in this study. Participants were nursing students interning in the otorhinolaryngology department of a Grade A tertiary hospital in Deyang City, enrolled through convenience sampling. Students who completed clinical internship from April 2024 to December 2024 were assigned to the control group, while those interning from January 2025 to August 2025 were assigned to the experimental group. This study involved a total of 86 subjects, with 43 interns in each group. The experimental group received the “7E + Ideological and Political Education” teaching model, while the control group received the traditional clinical teaching methods.

### Sample size calculation

The sample size was calculated using the formula for comparing the means of two independent samples based on the study design. Critical thinking ability was employed as the primary outcome measure. According to prior research, the critical thinking ability score of the control group was 295.28 ± 15.86, while the score of the experimental group was 308.75 ± 18.05. The significance level was set at α = 0.05 with statistical power of 0.90(1-β = 0.90). According to the formula:

$$\mathrm n=\frac{\left({\mathrm Z}_a\;+\;{\mathrm Z}_\beta\right)^2\;2\mathrm\sigma^2}{\mathrm\delta^2}$$

The preliminary calculated sample size per group was 33. After accounting for a 10% loss to follow-up, the minimum required sample size was adjusted to 38 participants per group. To ensure stable statistical power, we ultimately recruited 43 subjects in each group.

### Ethical requirements

This study strictly adhered to ethical principles. Prior to implementation, the research purpose and procedures were clearly explained to both instructors and students. Prior to participation, all nursing students were provided with detailed information regarding the study’s aims and procedures. Written informed consent was then secured from each participant. The study emphasized that participation was entirely voluntary, data would be handled confidentially, and participants had the right to withdraw at any time without penalty. The study protocol was approved by the Deyang People’s Hospital Ethics Review Committee (Ethics Approval No.: 2025-04-020-K01).

### Research process

#### Control group

The control group received 4‑week clinical internship. The traditional teaching model was used. The chief preceptor conducted unified departmental orientation according to the internship plan, explaining departmental rules, regulations, and internship requirements. Each nursing student was assigned a fixed instructor to follow for learning and practice throughout their placement. Theoretical lectures were delivered once weekly, and case discussions were held once weekly.

#### Experimental group

The experimental group also underwent a 4-week clinical internship using the 7E learning cycle model integrated with ideological and political education elements. All clinical preceptors involved in the experimental group participated in a unified training session on the 7E model and its implementation prior to the study.

- ① Elicit (Daily, 40-60 min after morning ward rounds, 5days/week)
  - Given the variety of conditions in otorhinolaryngology, a competition points system (1 point per correct answer, with weekly top scorers receiving small rewards) was first used to guide interns to name common diseases encountered in the department, such as sudden sensorineural hearing loss, chronic rhinosinusitis, and laryngeal cancer. The instructor then provided 2-3 real clinical cases along with relevant images, endoscopy videos and nursing records to enhance interns’ understanding of professional diseases and special treatments. For example, when showing a case of postoperative laryngeal cancer, the instructor highlighted the patient’s breathing difficulties and communication barriers, prompting interns to consider the nurse’s role in alleviating patient distress. This approach stimulated interns’ learning interest while fostering a sense of responsibility. During explanations, concepts such as the spirit of prudence even when alone, patient rights, and privacy protection were interwoven to strengthen medical ethics education.
- ② Engage (skill operation training, twice weekly, 30-40 min per session)
  - In this stage, clinical instructors selected one typical departmental case per session, such as chronic rhinosinusitis requiring nasal irrigation, and demonstrated the corresponding nursing skill, emphasizing patient assessment, procedural steps and emergency responses. Following the demonstration, one intern was selected in rotation (ensuring each intern had at least one opportunity) to perform a return demonstration under direct supervision. The remaining interns observed the performance and were asked to identify both correct actions and areas needing improvement. During instruction, instructors demonstrated compassionate communication skills and integrated humanistic care into technical procedures, implicitly reinforcing the value of patient‑centered care.
- ③ Explore (Weekly case discussion, 20-30min per session, 4 sessions in total)
  - This stage involved organizing discussions on cases with potential ethical or communication disputes, such as a patient with laryngeal cancer refusing tracheostomy. At the start of each session, the instructor presented the case scenario, then interns had free discussion. Instructors encouraged interns to voluntarily share their analysis of the case from multiple perspectives, including nursing, ethics and communication. The instructor facilitated the discussion by posing probing questions, for example, “What would you do if the patient continued to refuse treatment?” or “How would you communicate with the patient’s family in this situation?” Every intern was required to voice at least one opinion during the session. The goal of this stage was to stimulate interns’ active thinking and expose them to the complexity and uncertainty inherent in real‑world clinical ethical dilemmas, rather than to arrive at a consensus.
- ④ Explain (Following the Explore session, 10-15min)
  - After the open discussion, the instructor provided an integrated summary, systematically sorting out the viewpoints proposed by interns, correcting factual misconceptions, supplementing key knowledge points that interns did not raise and highlighting core ethical principles. Through this process, interns were guided to reflect on the professional qualities that healthcare workers should possess. Unlike the preceding Explore stage which emphasized divergent thinking, this Explain stage provided convergent knowledge consolidation, ensuring that students left each session with a structured and actionable understanding of the case.
- ⑤ Elaborate (Biweekly situational simulation training, 60-90min/session)
  - The head instructor designed multiple case scenarios in advance. Two situational simulation exercises were conducted over the 4-week rotation. For example, the first scenario was “Post‑operative airway management for a laryngeal cancer patient”; the second was “Managing a conflict with a patient’s family due to prolonged waiting time.” Based on these scenarios, students independently planned situational simulations and created scripts. After the complete presentation, students evaluated each other and summarized shortcomings, including clinical accuracy, humanistic care, and communication skills. We hoped these activities would help cultivate awareness of teamwork, enhance self-directed learning ability, and create a more active classroom atmosphere. When writing scripts, students were required to reflect high professional ethics, sincere humanistic care, and natural and effective interpersonal communication methods.
- ⑥ Evaluate (At the end of Week 4)
  - At the end of Week 4, the chief preceptor carried out a comprehensive assessment for all interns, including a 50‑point theoretical test and a 50‑point practical skill examination. After the examination, interns were required to summarize and reflect on their learning, and the chief preceptor provided feedback on their internship performance.
- ⑦ Extend (At Week 3, 30-40 min)
  - The focus of this stage was to help interns broaden their clinical knowledge and gain a deeper understanding of professional spirit. To this end, the chief preceptor organized a debate on the topic: “Is nursing technique more important, or is nursing humanity more important?” Interns were given one week in advance to prepare their arguments using provided literature and their own clinical observations. The debate guided everyone to deeply consider how to build a more harmonious and effective nurse-patient relationship.

### Outcome evaluation

#### Assessment

Upon completion of the departmental internship, students underwent theoretical and clinical operational skill exit assessments using the same methods. The theoretical knowledge test included single-choice, multiple-choice, and short-answer questions, totaling 50 points. The clinical operational skill assessment required students to randomly select one specialized departmental operation, totaling 50 points.

#### Critical thinking ability

Critical thinking ability was measured with the Critical Thinking Disposition Inventory-Chinese Version (CTDI-CV), which was translated and revised by Peng Meici et al. [18]. This scale includes 7 dimensions, with a total score range of 70–420. Higher scores indicate stronger critical thinking ability. The Cronbach’s α of this scale is 0.9, indicating high internal consistency.

#### Self-directed learning ability

Self-directed learning ability was measured with the Self-Rating Scale for Self-Directed Learning (SRSSDL) compiled by Williamson [19] and revised into Chinese by Shen Wangqin et al. [20]. This scale primarily assesses five dimensions: self-directed learning awareness, learning strategies, learning behaviors, learning evaluation, and interpersonal skills. Higher scores indicate stronger self-directed learning ability. The Cronbach’s α of this scale ranges from 0.71 to 0.79.

#### Communication skills attitude

Communication attitudes were assessed with the Chinese version of the Communication Skills Attitude Scale (Ch-CSAS) [21]. The scale covers 26 items and adopts a 5-point Likert scoring system. It includes two subscales: the Positive Attitude Scale (PAS) and Negative Attitude Scale (NAS). The Cronbach’s α is 0.799 for PAS and 0.723 for NAS, with a test-retest reliability coefficient of *r* = 0.894 [22]. A higher PAS score indicates a more positive attitude toward learning communication skills, whereas a higher NAS score indicates a less positive attitude [23].

#### Nurse job esteem

Nursing students’ job esteem was measured with the Chinese version of the Nurse Job Esteem Scale (JES-HN). The scale was originally developed by Choi et al. [24] in 2020, and the sinicized and revised version translated by Shi Xiaopu et al. [25] was adopted in this study. The scale contains 25 items covering five dimensions: professional self-recognition, professional competence, social trust and respect, organizational respect and recognition, and professional authority and future value. All items adopt a 5-point Likert scoring system. Higher scores indicate stronger job esteem among nursing students. The scale shows excellent internal consistency, with an overall Cronbach’s α coefficient of 0.942.

### Statistical methods

SPSS 26.0 was used for data processing and statistical analysis, with *P* < 0.05 set as statistically significant.

#### Descriptive statistics

Normality tests were first conducted on the data from both groups. Measurement data conforming to a normal distribution were expressed as mean ± standard deviation; those not conforming were described using median and interquartile range. Count data were expressed as frequency (composition ratio).

#### Intergroup comparison

For measurement data conforming to a normal distribution, t-tests were used for analysis; for those not conforming to a normal distribution, the Mann-Whitney U test was used. For count data, chi-square tests were uniformly used for comparison.

#### Multiple comparison correction

A total of 24 independent hypothesis tests were conducted across the five outcome measures. To control the family-wise error rate, the Bonferroni correction was applied, with the adjusted significance level set at α’ = 0.05/24 = 0.0021. Only results with *P* < 0.0021 were considered statistically significant after correction.

## Results

### Comparison of general data

Before the intervention, a comprehensive analysis of the baseline data of the two groups of nursing students was conducted. The results showed no statistically significant differences between the two groups in terms of gender, age, place of origin, only-child status, pre-course grades in otorhinolaryngology nursing, and attitude towards specialized nursing (*P* > 0.05). This indicates that the two groups were comparable at the start of the study, making subsequent comparison results valuable (Table 1 for details).

**Table 1:** Comparison of General Information Between Two Groups of Nursing Students

Item	Control Group (*n* = 43)		Experimental Group (*n* = 43)	t or χ²
Age (years)		21.25 ± 1.18	21.45 ± 1.45	-0.540
Gender				0.245
Male		38	35	
Female		5	8	
Place of Origin				0.252
Rural		24	20	
Urban		19	23	
Whether an Only Child				1.678
Yes		15	17	
No		28	26	

### Comparison of theoretical exam scores and clinical operational skill scores (Table 2)

**Table 2:** Comparison of Theoretical Exam Scores and Clinical Operational Skill Exam Scores between the Two Groups of Nursing Students (x̄ ± s, points)

Time	Index	Group	xˉ±s	95% CI	t	*P*	Cohen’s d
Before intervention	Theoretical exam score	Experimental group	41.25 ± 3.72	40.14–42.36	6.372	0.217	0.33
		Control group	40.19 ± 2.49	39.45–40.93			
	Clinical operation skill exam score	Experimental group	41.59 ± 1.58	41.12–42.06	1.251	0.263	0.36
		Control group	41.06 ± 1.35	40.66–41.46			
	Total score	Experimental group	83.84 ± 3.79	82.70–84.98	9.731	0.073	0.71
		Control group	81.25 ± 3.51	80.19–82.31			
After intervention	Theoretical exam score	Experimental group	46.37 ± 1.36	45.96–46.78	3.043	< 0.001	2.22
		Control group	42.85 ± 1.72	42.33–43.37			
	Clinical operation skill exam score	Experimental group	42.15 ± 1.04	41.84–42.46	9.641	< 0.001	0.49
		Control group	41.41 ± 1.82	40.86–41.96			
	Total score	Experimental group	88.52 ± 2.24	87.85–89.19	13.350	< 0.001	1.91
		Control group	84.26 ± 2.12	83.60–84.92			

### Comparison of critical thinking ability

Scores for critical thinking ability were higher in the experimental group than in the control group (*P* < 0.001) (Table 3).

**Table 3:** Comparison of critical thinking ability between the two groups of nursing students (x̄ ± s, points)

Time	Dimension	Group	xˉ±s	95% CI	t	*P*	Cohen’s d
Before intervention	Truth-Seeking	Experimental group	36.09 ± 4.58	34.68–37.50	0.473	0.661	0.097
		Control group	36.52 ± 4.31	35.20–37.84			
	Open-Mindedness	Experimental group	34.18 ± 4.23	32.88–35.48	0.318	0.701	0.070
		Control group	34.57 ± 4.35	33.24–35.90			
	Analyticity	Experimental group	36.36 ± 4.65	34.93–37.79	1.061	0.271	0.051
		Control group	36.13 ± 4.31	34.81–37.45			
	Systematicity	Experimental group	35.82 ± 5.62	34.10–37.54	0.784	0.438	0.080
		Control group	35.38 ± 5.31	33.77–36.99			
	Self-Confidence	Experimental group	38.55 ± 5.92	36.74–40.36	0.296	0.751	0.052
		Control group	38.23 ± 6.30	36.30–40.16			
	Inquisitiveness	Experimental group	42.64 ± 6.05	40.79–44.49	0.287	0.761	0.040
		Control group	42.87 ± 5.46	41.20–44.54			
	Maturity	Experimental group	34.33 ± 4.98	32.80–35.86	0.375	0.709	0.006
		Control group	34.36 ± 5.05	32.81–35.91			
	Total score	Experimental group	257.97 ± 20.12	251.81–264.13	0.416	0.683	0.004
		Control group	258.06 ± 21.44	251.49–264.63			
After intervention	Truth-Seeking	Experimental group	37.61 ± 6.35	35.67–39.55	8.173	< 0.001	0.043
		Control group	37.31 ± 7.53	35.01–39.61			
	Open-Mindedness	Experimental group	34.67 ± 6.16	32.79–36.55	9.510	< 0.001	0.150
		Control group	33.75 ± 6.05	31.90–35.60			
	Analyticity	Experimental group	38.67 ± 7.08	36.52–40.82	6.973	< 0.001	0.401
		Control group	36.03 ± 6.07	34.18–37.88			
	Systematicity	Experimental group	38.48 ± 7.83	36.10–40.86	6.247	< 0.001	0.277
		Control group	36.31 ± 7.56	34.00–38.62			
	Self-Confidence	Experimental group	41.13 ± 6.45	39.17–43.09	5.936	< 0.001	0.316
		Control group	38.93 ± 7.56	36.62–41.24			
	Inquisitiveness	Experimental group	44.95 ± 6.72	42.90–47.00	5.957	< 0.001	0.293
		Control group	43.55 ± 8.51	40.94–46.16			
	Maturity	Experimental group	38.78 ± 5.43	37.12–40.44	8.064	< 0.001	0.452
		Control group	35.83 ± 7.64	33.49–38.17			
	Total score	Experimental group	274.29 ± 32.32	264.42–284.16	10.027	< 0.001	0.368
		Control group	261.71 ± 36.53	250.54–272.88			

### Comparison of self-directed learning ability

We found that across all dimensions of self-directed learning ability—including learning awareness, learning strategies, learning behaviors, learning evaluation, and interpersonal skills—scores for the experimental group were significantly higher than those of the control group, and the total score was also significantly better (*P* < 0.001) (Table 4).

**Table 4:** Comparison of self-directed learning ability between the two groups of nursing students (x̄ ± s, points)

Time	Dimension	Group	xˉ±s	95% CI	t	*P*	Cohen’s d
Before intervention	Learning Awareness	Experimental	41.19 ± 5.15	39.65–42.73	3.492	0.211	2.33
		Control	30.03 ± 4.13	28.80–31.26			
	Learning Strategies	Experimental	39.39 ± 6.01	37.59–41.19	9.393	< 0.001	1.75
		Control	28.69 ± 6.12	26.85–30.53			
	Learning Behavior	Experimental	40.12 ± 4.31	38.83–41.41	9.448	< 0.001	2.47
		Control	29.68 ± 4.12	28.45–30.91			
	Learning Evaluation	Experimental	42.33 ± 3.32	41.34–43.32	9.641	0.361	3.57
		Control	29.67 ± 3.81	28.53–30.81			
	Interpersonal Skills	Experimental	40.31 ± 3.59	39.24–41.38	8.350	< 0.001	2.86
		Control	30.19 ± 3.26	29.21–31.17			
	Total Score	Experimental	203.34 ± 15.16	198.81–207.87	8.043	< 0.001	3.84
		Control	148.26 ± 13.19	144.34–152.18			
After intervention	Learning Awareness	Experimental	42.85 ± 5.72	41.14–44.56	8.043	< 0.001	2.34
		Control	30.57 ± 4.36	29.30–31.84			
	Learning Strategies	Experimental	43.47 ± 4.61	42.09–44.85	9.393	< 0.001	2.77
		Control	31.21 ± 4.25	29.97–32.45			
	Learning Behavior	Experimental	44.35 ± 4.31	43.06–45.64	9.448	< 0.001	2.64
		Control	30.89 ± 5.72	29.18–32.60			
	Learning Evaluation	Experimental	44.41 ± 4.82	42.98–45.84	9.641	< 0.001	2.61
		Control	30.15 ± 6.04	28.34–31.96			
	Interpersonal Skills	Experimental	43.81 ± 5.12	42.30–45.32	8.350	< 0.001	2.59
		Control	30.53 ± 5.24	28.99–32.07			
	Total Score	Experimental	218.89 ± 24.58	211.64–226.14	8.043	< 0.001	2.60
		Control	153.35 ± 25.61	145.82–160.88			

### Comparison of communication skills attitude

The experimental group’s score on the positive attitude scale (PAS) was significantly higher than that of the control group (*P* < 0.05), while their score on the negative attitude scale (NAS) was significantly lower (*P* < 0.05). (Table 5)

**Table 5:** Comparison of Communication Skills Attitude between the Two Groups of Nursing Students (x̄ ± s, points)

Time	Index	Group	xˉ±s	95% CI	t	*P*	Cohen’s d
Before intervention	PAS Total Score	Experimental	50.73 ± 5.01	49.24–52.22	2.457	0.207	2.27
		Control	41.17 ± 2.82	40.33–42.01			
	NAS Total Score	Experimental	33.65 ± 4.37	32.35–34.95	5.293	0.428	0.06
		Control	33.93 ± 5.11	32.41–35.45			
After intervention	PAS Total Score	Experimental	56.33 ± 2.56	55.57–57.09	9.413	< 0.05	4.51
		Control	43.25 ± 3.12	42.32–44.18			
	NAS Total Score	Experimental	30.12 ± 1.41	29.70–30.54	8.481	< 0.05	1.11
		Control	32.45 ± 2.51	31.70–33.20			

### Comparison of job esteem

Scores for the experimental group nursing students were higher than those of the control group across all five dimensions of job esteem (*P* < 0.001) (Table 6).

**Table 6:** Comparison of Job Esteem between the Two Groups of Nursing Students (x̄ ± s, points)

Time	Dimension	Group	xˉ±s	95% CI	t	*P*	Cohen’s d
Before intervention	Professional Competence	Experimental	30.63 ± 6.28	28.75–32.51	6.241	< 0.001	0.35
		Control	28.61 ± 5.41	26.99–30.23			
	Professional Self-Recognition	Experimental	12.67 ± 2.75	11.85–13.49	2.826	< 0.001	0.60
		Control	10.69 ± 3.81	9.55–11.83			
	Social Trust & Respect	Experimental	10.09 ± 2.16	9.44–10.74	4.503	< 0.001	0.16
		Control	10.48 ± 2.81	9.64–11.32			
	Organizational Respect & Recognition	Experimental	13.73 ± 3.61	12.65–14.81	2.751	< 0.001	0.43
		Control	12.29 ± 3.16	11.35–13.23			
	Professional Authority & Future Value	Experimental	12.14 ± 3.57	11.07–13.21	3.842	< 0.001	0.66
		Control	10.02 ± 2.72	9.21–10.83			
	Total Score	Experimental	79.26 ± 17.73	73.95–84.57	6.422	< 0.001	0.43
		Control	72.09 ± 13.83	67.96–76.22			
After intervention	Professional Competence	Experimental	34.22 ± 5.23	32.66–35.78	7.352	< 0.001	0.70
		Control	30.73 ± 4.61	29.35–32.11			
	Professional Self-Recognition	Experimental	13.89 ± 3.17	12.94–14.84	6.315	< 0.001	1.20
		Control	11.21 ± 2.59	10.43–11.99			
	Social Trust & Respect	Experimental	10.44 ± 2.15	9.79–11.09	4.835	< 0.001	0.21
		Control	10.90 ± 2.20	10.23–11.57			
	Organizational Respect & Recognition	Experimental	14.22 ± 3.27	13.24–15.20	5.137	< 0.001	0.60
		Control	12.52 ± 2.41	11.80–13.24			
	Professional Authority & Future Value	Experimental	12.67 ± 4.29	11.39–13.95	2.051	< 0.001	0.55
		Control	10.39 ± 3.48	9.35–11.43			
	Total Score	Experimental	85.44 ± 18.11	79.99–90.89	8.352	< 0.001	0.57
		Control	75.75 ± 15.31	71.13–80.37			

### Students’ reflective feedback

In their weekly reflective journals and group discussion transcripts, students in the experimental group frequently mentioned that the case-based discussions and situational simulations helped them understand the importance of humanistic care and professional ethics in clinical practice. Several students noted that role-playing scenarios deepened their empathy toward patients with sensory dysfunctions, while others reported increased confidence in communicating with anxious patients and their families.

Specifically, many students reflected that the authentic clinical cases presented during the Elicit phase made them more aware of patients’ psychological distress. Some mentioned that the debate on “nursing technique versus nursing humanity” prompted them to reconsider the essence of the nursing profession. A number of students also expressed that peer feedback during the Elaborate phase was particularly valuable in helping them identify blind spots in their communication approaches.

## Discussion

This study innovatively integrates the 7E Learning Cycle Model with ideological and political education, constructing an integrated teaching intervention based on a 4-week clinical internship in otorhinolaryngology nursing and conducting a controlled trial against the traditional preceptorship model. The research results demonstrate that compared with traditional lecture-based preceptorship, the integrated 7E and ideological-political education teaching model achieves significant improvements across four training dimensions and presents prominent teaching advantages, which can provide practical evidence and innovative ideas for the reform of specialized nursing clinical teaching.

### The integrated 7E and ideological-political education teaching model promotes the in-depth internalization of otorhinolaryngology nursing knowledge and operational skills

Quantitative intergroup comparisons reveal that students in the experimental group obtained markedly higher scores in theoretical assessments and clinical skill evaluations than those in the control group (*P* < 0.001). Rather than merely presenting intergroup differences, this section analyzes the internal mechanisms through which the model improves professional competencies based on the progressive seven-stage teaching framework. The teaching process starts with the Elicit phase, where authentic ENT clinical cases and endoscopic imaging materials activate students’ prior knowledge reserves and stimulate their intrinsic learning motivation, overcoming the passive learning drawbacks of traditional one-way lecture-based teaching. In the subsequent Engage and Explore phases, students are guided to independently analyze complex clinical problems instead of receiving standardized answers passively, so as to build a systematic knowledge framework on their own. During the Explain and Elaborate phases, group seminars, clinical scenario simulations, result presentations and mutual feedback establish a transformation channel for theoretical knowledge application. Abstract textbook knowledge is translated into standardized operational procedures combined with discipline-specific clinical scenarios of otorhinolaryngology, enabling genuine knowledge transfer rather than superficial rote memorization. This study optimizes the teaching scheme tailored to the characteristics of otorhinolaryngology, enriches empirical research on the application of the 7E model in nursing education, and verifies that embedding discipline-specific clinical scenarios can amplify the competency cultivation effect of the model on practical skills.

### The integrated 7E and ideological-political education teaching model realizes the coordinated integration of knowledge instruction and value guidance

A comprehensive evaluation covering critical thinking, self-directed learning, doctor-patient communication and nursing professional identity was carried out in this study. All indicators reflect better performance among students in the experimental group, which verifies that the 7E cyclic teaching framework can serve as an integration carrier to connect professional skill teaching with ideological and political value guidance, addressing the long-standing dilemma of the separation of professional teaching and moral education in specialized nursing training.

### Inquiry-oriented teaching design throughout the cycle improves students’ comprehensive cognitive literacy

Students exhibited remarkable improvements in critical thinking and self-directed learning abilities (Tables 3 and 4), which can be mainly attributed to the targeted inquiry-based teaching tasks arranged in the Explore and Extend phases. In the Explore phase, high-risk ENT nursing cases are adopted for group discussions, requiring students to compare different intervention plans and identify potential nursing safety hazards. The Extend phase introduces debates on medical ethics, guiding students to analyze clinical nursing dilemmas from multiple perspectives including medical ethics, patient rights and institutional management regulations. Such teaching designs break the rigid thinking mode centered on single standard answers and cultivate students’ capabilities of multi-angle analysis, evidence-based decision-making and active exploration. Unlike traditional teaching that only arranges one-time speculative activities, the integrated 7E model embeds reflective and divergent thinking tasks throughout the entire internship cycle, turning dialectical analysis into a regular learning habit. Meanwhile, the full-cycle learning mode featuring independent clinical problem-solving, collaborative group inquiry and post-practice review can train students to independently formulate learning objectives, dynamically adjust learning strategies and summarize clinical experience, laying a solid foundation for their core literacy supporting sustainable lifelong career development after graduation.

### Phased embedded ideological and political design enables implicit cultivation of professional values

The core innovation of this model lies in its phased integration of ideological and political education elements into routine clinical teaching activities, rather than treating them as standalone lectures or add-on modules. Through scenario-based role-playing and structured case discussions, students were prompted to consider not only the technical aspects of nursing procedures but also the emotional and ethical dimensions of patient care. For instance, when managing a post-laryngectomy patient with communication difficulties, students had to work out not just airway clearance techniques, but also alternative communication methods and emotional support strategies. This design appeared to help students connect clinical reasoning with humanistic awareness in a concrete, task-oriented manner, which may partly explain the improved job esteem and communication attitude scores observed in the experimental group (Tables 5 and 6).

Mechanistically, the hierarchical structure of the 7E model—moving from basic knowledge elicitation to complex scenario elaboration—provided natural entry points for embedding ethical discussions without disrupting the flow of clinical skill training. Unlike approaches that segregate professional content from values education, this integrated format allowed students to encounter ethical tensions (e.g., patient refusal of treatment, family conflicts over prolonged waiting times) as inherent parts of clinical practice rather than as abstract hypotheticals. By repeatedly engaging with such tensions across the Explore and Elaborate phases, students had opportunities to practice balancing clinical priorities with patient preferences, a core competency required in real-world otorhinolaryngology nursing. Whether this repeated exposure translates into lasting behavioral change in future practice remains to be determined.

Additionally, students’ reflective journals offered some anecdotal support for these quantitative trends. Several students noted that role-playing a patient with sudden hearing loss made them more conscious of the anxiety caused by sensory impairment, and that the debate on “technique versus humanity” pushed them to articulate and defend their own value positions. However, these reflections were collected as part of routine teaching documentation rather than through a formal qualitative research protocol. Therefore, they should be interpreted as illustrative feedback that aligns with the statistical findings, not as independent evidence of causal mechanisms. Future studies with structured interviews or observational methods would be needed to more rigorously examine how students actually internalize professional values during clinical rotations.

## Research limitations

Several limitations of this exploratory study should be acknowledged. First, this was a single-center, quasi-experimental study employing convenience sampling with a relatively small sample size. The absence of multi-center randomization limits the generalizability of our findings, and the modest sample may render the results susceptible to confounding by individual baseline competencies. Second, the follow-up period was confined to the immediate post-rotation assessment, without extended observation. Consequently, the long-term sustainability of the intervention’s effects on professional values and autonomous learning behaviors remains undetermined. Third, the outcome measures relied predominantly on self-reported scales for constructs such as critical thinking and professional identity, which are vulnerable to social desirability bias. The lack of corroborating objective data such as clinical instructor evaluations or direct behavioral observations further limits the robustness of our conclusions. Therefore, future research can adopt multi-center randomized designs, extend follow-up durations, and combine self-reported questionnaires with objective evaluation indicators to produce more credible and generalizable evidence.

## Summary

The “7E Learning Cycle - Ideological and Political Education in Curriculum” model demonstrated good practical results in otorhinolaryngology nursing teaching. This model not only helped students more solidly master theoretical knowledge but also significantly improved their clinical operational skills, highlighting its outstanding advantages in cultivating professional competence.

More importantly, this model positively impacted students’ comprehensive qualities such as critical thinking, autonomous learning, communication, collaboration, and professional identity. It truly achieved “teaching professional knowledge while also cultivating comprehensive human qualities.” Leveraging the step-by-step teaching design of the 7E learning cycle, ideological and political elements such as humanistic care and professional ethics were no longer rigid add-ons but were naturally integrated into the entire teaching process. Thus, value shaping and ability enhancement were achieved simultaneously.

This model aligns with the current need for cultivating high-quality nursing talent and provides clinical teachers with a systematic, clear, easy-to-operate, and effective teaching method worthy of further promotion and application.

## Data Availability

All data generated or analysed during this study are included in this published article. The datasets used and/or analysed during the current study are available from the corresponding author on reasonable request.

## Declarations

This study was conducted in accordance with the principles of the Declaration of Helsinki. Written informed consent was obtained from all participants prior to their inclusion in the study.
